# Sugammadex versus neostigmine for neuromuscular blockade reversal in outpatient surgeries: A randomized controlled trial to evaluate efficacy and associated healthcare cost in an academic center

**DOI:** 10.3389/fmed.2022.1072711

**Published:** 2022-12-08

**Authors:** Juan Fiorda Diaz, Marco Echeverria-Villalobos, Alan Esparza Gutierrez, Olufunke Dada, Nicoleta Stoicea, Wiebke Ackermann, Mahmoud Abdel-Rasoul, Jarrett Heard, Alberto Uribe, Sergio D. Bergese

**Affiliations:** ^1^Department of Anesthesiology, The Ohio State University Wexner Medical Center, Columbus, OH, United States; ^2^College of Medicine, The Ohio State University Wexner Medical Center, Columbus, OH, United States; ^3^Department of Anesthesiology, University of Toledo, Toledo, OH, United States; ^4^Department of Biomedical Informatics, Center for Biostatistics, The Ohio State University Wexner Medical Center, Columbus, OH, United States; ^5^Department of Anesthesiology, Stony Brook University, Stony Brook, NY, United States

**Keywords:** neuromuscular blockade, neuromuscular blocking agents, rocuronium, neostigmine, cost-benefit analysis, sugammadex

## Abstract

**Introduction:**

Neuromuscular blockade is an essential component of the general anesthesia as it allows for a better airway management and optimal surgical conditions. Despite significant reductions in extubation and OR readiness-for-discharge times have been associated with the use of sugammadex, the cost-effectiveness of this drug remains controversial. We aimed to compare the time to reach a train-of-four (TOF) response of ≥0.9 and operating room readiness for discharge in patients who received sugammadex for moderate neuromuscular blockade reversal when compared to neostigmine during outpatient surgeries under general anesthesia. Potential reduction in time for OR discharge readiness as a result of sugammadex use may compensate for the existing cost-gap between sugammadex and neostigmine.

**Methods:**

We conducted a single-center, randomized, double arm, open-label, prospective clinical trial involving adult patients undergoing outpatient surgeries under general anesthesia. Eligible subjects were randomized (1:1 ratio) into two groups to receive either sugammadex (Groups S), or neostigmine/glycopyrrolate (Group N) at the time of neuromuscular blockade reversal. The primary outcome was the time to reverse moderate rocuronium-induced neuromuscular blockade (TOF ratio ≥0.9) in both groups. In addition, post-anesthesia care unit (PACU)/hospital length of stay (LOS) and perioperative costs were compared among groups as secondary outcomes.

**Results:**

Thirty-seven subjects were included in our statistical analysis (Group S= 18 subjects and Group N= 19 subjects). The median time to reach a TOF ratio ≥0.9 was significantly reduced in Group S when compared to Group N (180 versus 540 seconds; *p* = 0.0052). PACU and hospital LOS were comparable among groups. Postoperative nausea and vomiting was the main adverse effect reported in Group S (22.2% versus 5.3% in Group N; *p* = 0.18), while urinary retention (10.5%) and shortness of breath (5.3%) were only experienced by some patients in Group N. Moreover, no statistical differences were found between groups regarding OR/anesthesia, PACU, and total admission costs.

**Discussion:**

Sugammadex use was associated with a significantly faster moderate neuromuscular blockade reversal. We found no evidence of increased perioperative costs associated with the use of sugammadex in patients undergoing outpatient surgeries in our academic institution.

**Clinical trial registration:**

[https://clinicaltrials.gov/] identifier number [NCT03579589].

## Introduction

Neuromuscular blockade is an essential component of general anesthesia as it allows for better airway management and surgical conditions (1). Nevertheless, the use of neuromuscular blocking agents (NMBAs) has been associated with higher risk of perioperative pulmonary complications, mostly due to postoperative residual neuromuscular blockade (2). After the introduction of quantitative neuromuscular monitoring devices to assess the train-of-four (TOF) ratio in the ’70s, subjective clinical parameters used for neuromuscular blockade monitoring progressively became obsolete (3).

Residual neuromuscular blockade is defined as a TOF ratio < 0.9 and results from incomplete reversal after using NMBAs (4, 5). The reported incidence of residual neuromuscular blockade is highly variable among trials, being identified in up to 40% of patients receiving non-depolarizing NMBAs (4, 6). The level of neuromuscular blockade varies among surgeries. Most non-abdominal surgical procedures are performed under moderate neuromuscular blockade (<2 responses in the TOF). In contrast, major abdominal surgeries may require the use of deep neuromuscular blockade defined as the presence of no twitches after TOF stimulation and only 1–2 post-tetanic count (PTC) responses during post-tetanic stimulation (7).

The use of deep neuromuscular blockade may improve working surgical conditions during major abdominal procedures and allow for significant reductions in insufflation pressures during laparoscopic surgeries. However, deep neuromuscular blockade has been also associated with prolonged postoperative muscle paralysis, residual neuromuscular blockade, hypoxemia and atelectasis (1, 8–11). These postoperative complications may prolong clinical recovery and post-anesthesia care unit (PACU) length of stay (LOS) with a subsequent increase in the overall cost to patients.

Sugammadex is a cyclodextrin with a novel mechanism of action and widely reported efficacy for neuromuscular blockade reversal (12, 13). Sugammadex encapsulates the rocuronium molecule creating a stable complex that is removed by the glomeruli, bypassing the hepatobiliary metabolic pathway (14). The dose of 2 mg/kg is recommended for the reversal of a moderate rocuronium- or vecuronium-induced neuromuscular blockade. Likewise, a dose of 4–8 mg/kg is effective for the reversal of deep neuromuscular blockade and a single dose of 16 mg/kg is advisable to reverse the neuromuscular blockade within 3 min after the administration of 1.2 mg/kg of rocuronium (15).

The use of sugammadex has been linked to a significant reduction in the time for extubation and operating room (OR) readiness for discharge. However, its cost-effectiveness remains unclear (16). In a systematic review, Paton et al. mentioned the lack of evidence concerning sugammadex cost and “efficient use of resources.” The review discussed the cost-effectiveness of the drug based on the United Kingdom practice where OR staff time was evaluated at £4.44 per minute (17). Similarly, the cost of sugammadex has been identified as one of the main limiting factor for its use in the United States (18).

Reported evidence on the incidence of postoperative pulmonary complications, residual neuromuscular blockade, postoperative nausea and vomiting (PONV), and PACU LOS after the use of sugammadex is highly variable (19).

In our study, we aimed to compare the time to reach a TOF response ≥ 0.9 and OR readiness for discharge after sugammadex administration for moderate neuromuscular blockade reversal when compared to neostigmine in outpatient surgeries under general anesthesia. We hypothesized that the administration of sugammadex for the reversal of moderate neuromuscular blockade will be associated with a significant reduction in time to reach a TOF response ≥ 0.9 and OR readiness for discharge when compared to neostigmine in patients undergoing outpatient surgeries at The Ohio State University Wexner Medical Center. In addition, we considered that the reduction in time to OR readiness for discharge will compensate for the existing cost-gap between sugammadex and neostigmine.

## Materials and methods

Local Institutional Review Board approval (Office of Responsible Research Practices. Protocol #2018H0102) and ClinicalTrials.gov registration number (NCT03579589) were obtained before starting this clinical trial. Patients provided written informed consent before study participation.

### Study design

We conducted a single-center, randomized, double-arm, open-label, prospective clinical trial involving adult patients undergoing outpatient surgeries at The Ohio State University Wexner Medical Center (OSUWMC) between August 2018 and April 2019. Adult patients (≥18 years old), American Society of Anesthesiologist (ASA) physical status I, II, or III undergoing outpatient surgeries (i.e., laparoscopic cholecystectomy, laparoscopic hernia repair, and laparoscopic appendectomy) under general anesthesia, and requiring rocuronium-induced neuromuscular blockade were included in the study. Prisoners, pregnant women, and patients with significant pre-existent clinical conditions such as chronic obstructive pulmonary disease (COPD), chronic kidney disease (CKD), and neuromuscular or neurodegenerative diseases were excluded.

### Randomization

Subjects were randomized on a 1:1 ratio into 2 groups: Group S (sugammadex) and group N (neostigmine/glycopyrrolate) by using the automated Research Electronic Data Capture (REDCap) system. Moreover, randomization was completed at the time of neuromuscular blockade reversal in order to avoid potential bias on intraoperative NMBAs dosage and administration.

### Study procedures

Demographics, medical history, physical examination, laboratory results (including pregnancy test), and prior medications were documented. Midazolam was administered for premedication as needed in all patients. Standard monitoring including 5-lead electrocardiogram (ECG), non-invasive blood pressure (NIBP) and pulse oximetry, was applied on arrival to the OR. Moreover, neuromuscular transmission was measured based on TOF stimulation of the ulnar nerve using the MechanoSensor (GE Healthcare^®^). Induction of general anesthesia consisted of pre-oxygenation (inspired fraction of oxygen 100% for at least 5 full Tidal Volume) and a drug sequence of intravenous (IV) lidocaine (0.5–1 mg/Kg), propofol (1–2 mg/kg), IV fentanyl (1–2 μg/Kg), rocuronium (0.3–0.7 mg/kg), and sevoflurane (0.5–1 of the minimum alveolar concentration or MAC). Total body weight (TBW) was used to calculate total dosage. Ulnar nerve stimulation device was calibrated and subsequently initiated after propofol and before rocuronium administration. Balanced anesthesia with sevoflurane (0.5–1.0 MAC) was administered for maintenance.

Moderate neuromuscular blockade was achieved after IV rocuronium (0.3–0.7 mg/kg) and monitored every 5 min using the TOF response. Supplemental doses of rocuronium were administered at discretion of the anesthesia provider to maintain an adequate moderate neuromuscular blockade (<2 TOF responses). Neuromuscular blockade reversal was administered after peritoneal (port sites) closure based on the TOF responses and group randomization as follows:

– Group S: sugammadex 2 mg/Kg of TBW when spontaneous recovery had reached a second twitch after TOF, or 4 mg/Kg of TBW when spontaneous recovery showed no twitch responses to TOF stimulation but between 1 and 2 PTC.
– Group N: neostigmine 50 μg. Kg^–1^ of TBW once spontaneous recovery had reached the fourth twitch after TOF stimulation, in accordance with our institutional standard procedures and published literature (20, 21).

Once neuromuscular blockade reversal was administered, the TOF responses were assessed every 30 seconds during the first 3 min and every minute afterward until a TOF ≥ 0.9 was obtained. Subsequently, the times from neuromuscular reversal administration to a TOF ratio ≥ 0.9, extubation and OR readiness-for-discharge were collected at the end of surgery. In addition, the time from peritoneal closure (port sites) to OR readiness-for-discharge was documented.

#### Postoperative procedures (post-anesthesia care unit)

On PACU arrival, all subjects received 2–3 liters per minute (L.min^–1^) of supplementary oxygen through a nasal cannula and standard monitoring was applied. Length of PACU stay (Phase I, defined as PACU LOS in minutes), length of hospital stay (Phase II, stepdown from PACU to the general floor or hospital discharge) and significant adverse events (e.g., bradycardia, anaphylaxis, nausea and vomiting, and hypotension) were collected.

### Perioperative costs

Uniform Billing (UB-04) hospital forms were retrieved after hospital discharge in order to obtain the OR, anesthesia and recovery services related costs from all subjects.

### Sample size and statistical analysis

The sample size was determined based on the primary outcome (time to reach a TOF response ≥ 0.9). A total of 40 patients would provide more than 90% power to detect a difference of 360 seconds. from drug administration to extubation, assuming standard deviations of 0.8 and 6.9 in the Sugammadex and control groups, respectively, at a 5% type I error rate (22). Summary statistics for continuous variables are reported as means (standard deviations) or medians [inter-quartile ranges] and as frequencies (percentages) for categorical variables. We compared the study groups using Student’s *t*-tests or Wilcoxon Rank-Sum tests for continuous variables, and chi-squared or Fisher’s exact tests for categorical variables. For secondary outcomes between study groups comparing time from peritoneal closure (port sites) to patient readiness for OR readiness–for-discharge to PACU we used the Student’s *t*-tests at the 5% type I error rate. All analyses were conducted using SAS version 9.4 (SAS Institute, Cary, NC, USA).

## Results

**FIGURE 1:**
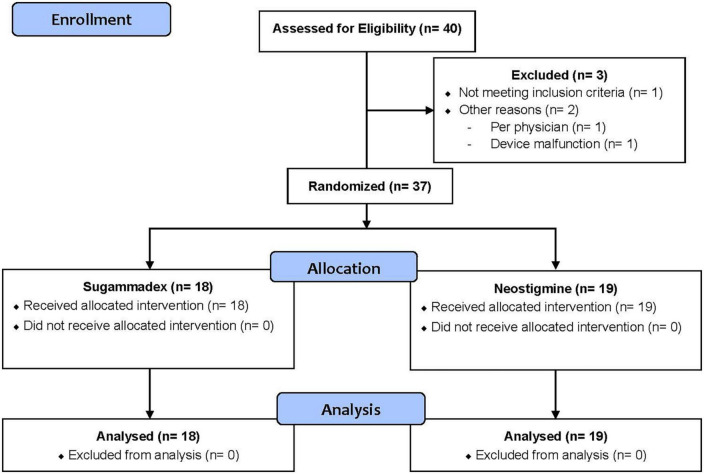
Consort flow diagram.

A total of 40 patients (*n* = 40) were enrolled in this trial. However, three patients (*n* = 3) were excluded from the analysis for the following reasons: did not meet inclusion/exclusion criteria (*n* = 1), physician provider discretion (*n* = 1) and device malfunction (*n* = 1). Therefore, 37 patients (*n* = 37) were included in our statistical analysis. Of these, 18 patients (*n* = 18) were randomized to Group S and 19 patients (*n* = 19) to Group N. Figure 1 displays our CONSORT flow diagram (23).

### Study population: Demographic and baseline characteristics

Demographic and baseline characteristics including gender, age, race, ASA physical status, and preoperative diagnosis were similar between groups. The median age was 48 [44, 65] years old in Group N and 56.5 [47, 63] years old in Group S (*p* = 0.71). Moreover, 58% of patients in Group N were males, whereas 61% in Group S were females. The median body mass index (BMI) was slightly higher in Group S when compared to Group N (32.5 versus 28.2 kg/m^2^, respectively; *p* = 0.06). Cholecystitis/calculus of gallbladder was the most common preoperative diagnosis in both groups (Table 1).

**TABLE 1:** Demographics and baseline variables.

Variable	Overall (*n* = 37)	Neostigmine (*n* = 19)	Sugammadex (*n* = 18)	*P*-value
Age, years, median [IQR]	56 [45, 63]	48 [44, 65]	56.5 [47, 63]	0.71
Gender, male, *n* (%)	18 (49%)	11 (58%)	7 (39%)	0.25
Gender, female, *n* (%)	19 (51%)	8 (42%)	11 (61%)	
**Race, mean (SD)**				
White	30 (81%)	16 (84%)	14 (78%)	
African-American	5 (14%)	2 (11%)	3 (17%)	0.86
Other	2 (5%)	1 (5%)	1 (6%)	
Height, m, median [IQR]	1.7 [2, 1.8]	1.7 [2, 1.9]	1.7 [2, 1.7]	0.27
Weight, kg, median [IQR]	95.3 [80, 102.5]	88.5 [79, 100.9]	96.2 [86, 103.9]	0.47
BMI, kg/m^2^, median [IQR]	30.4 [28, 34.8]	28.2 [27, 33.8]	32.5 [29, 39.1]	0.06
ASA classification, I/II/III, n	2/25/10	2/12/5	0/13/5	0.37
**Preoperative diagnosis *n* (%)**				
Calculus of gallbladder/cholecystitis	19 (51.4)	9 (47.4)	10 (55.6)	
Inguinal hernia	11 (29.7)	8 (42.1)	3 (16.7)	0.10
Ventral hernia	6 (16.2)	1 (5.3)	5 (27.8)	
Appendix condition (mass, appendicitis)	1 (2.7)	1 (5.3)	0 (0.00)	

IQR, interquartile range; SD, standard deviation.

### Primary and secondary outcomes

The median time to reach a TOF response ≥ 0.9 was significantly reduced in Group S when compared to Group N (180 versus 540 s; *p* = 0.0052). The median time between incision closure and extubation was slightly shorter in Group S when compared with Group N (4.5 [2, 6] versus 7 [5, 8] min; *p* = 0.26). In contrast, the median time elapsed from neuromuscular blockade reversal administration to extubation was slightly shorter in the Group S when compared to Group N (10.5 [11, 16] versus 13 [11, 16] min; *p* = 0.19). Overall, the median time elapsed from neuromuscular blockade reversal administration to OR readiness-for-discharge was similar between Group S and Group N (14 [9, 16] versus 15 [14, 18] min, respectively; *p* = 0.14) (Table 2).

**TABLE 2:** Perioperative variables.

Variable	Overall (*n* = 37)	Neostigmine (*n* = 19)	Sugammadex (*n* = 18)	*P*-value
**Procedure performed n (%)**				
Inguinal hernia repair	11 (29.7)	8 (42.1)	3 (16.7)	
Cholecystectomy	19 (51.4)	9 (47.4)	10 (55.6)	0.10
Appendectomy	1 (2.7)	1 (5.3)	0 (0.00)	
Ventral hernia repair	6 (16.2)	1 (5.3)	5 (27.8)	
Length of anesthesia, min, median [IQR]	96 [80, 127]	98 [80, 132]	94.5 [74, 117]	0.55
Length of surgery, min, median [IQR]	61 [51, 90]	61 [51, 93]	61.5 [51, 78]	0.84
Time to TOF ≥ 0.9, s	*missing = 11 240 [150, 420]	*missing = 10 540 [360, 600]	*missing = 1 180 [150, 300]	0.0052
Incision closure to extubation time, min, median [IQR]	6 [3,8]	7 [5,8]	4.5 [2,6]	0.26
IP Administration to extubation time, min, median [IQR]	12 [8, 15]	13 [11, 16]	10.5 [7, 15]	0.19
IP Administration to anesthesia readiness, min, median [IQR]	15 [12, 17]	15 [14, 18]	14 [9, 16]	0.14
Phase I duration, min, median [IQR]	60 [44, 90]	56 [45, 84]	63.5 [44, 118]	0.31
Phase II duration, min, median [IQR]	133 [85, 213]	166 [102, 245]	118 [83, 175]	0.11
Total time hospitalization, min, median [IQR]	510 [415, 604]	543 [430, 625]	466.5 [404, 548]	0.32

IQR, interquartile range; SD, standard deviation; TOF, train of four. IP, investigational product (neostigmine/glycopyrrolate or sugammadex).

*Extubation was performed before TOF ratio ≥ 0.9 at anesthesia care provider’s discretion.

Cholecystectomy was the most common procedure performed in both groups (51.4%). There were no statistically significant differences in median length of anesthesia or surgery between groups (*p* = 0.55 and *p* = 0.84, respectively). The median PACU LOS (Phase I) was similar in both groups (56 [45, 84] min for Group N and 63 [44, 118] min for Group S; *p* = 0.31). Likewise, the median length of hospital stay (Phase II and/or hospitalization) was similar between groups (166 [102, 245] min for Group N versus 118 [83, 175] min for Group S; *p* = 0.11). In addition, the total time of hospitalization for Group N was 543 and 466.5 min for Group S (*p* = 0.32) (Table 2).

#### Perioperative complications

The overall incidence of adverse events was 21.6% and there were not statistical difference among groups. However, postoperative nausea and vomiting, urinary retention, and shortness of breath were the most common perioperative complications. Postoperative nausea and vomiting was experienced by 1 patient in Group N (5.3%) and 4 patients in Group S (22.2%); *p* = 0.18. Postoperative urinary retention was reported in 2 subjects from Group N (10.5%). These 2 subjects required 2 days of hospitalization until resolution. Lastly, shortness of breath was experienced by 1 subject in Group N (5.3%), requiring hospitalization for 24 h until resolution (Table 3).

**TABLE 3:** Postoperative complications.

Variables	Overall (*n* = 37)	Neostigmine (*n* = 19)	Sugammadex (*n* = 18)	*P*-value
Postoperative nausea and vomiting *n* (%)	5 (13.5)	1 (5.3)	4 (22.2)	0.18
Urinary retention *n* (%)	2 (5.4)	2 (10.5)	0 (0.0)	0.49
Shortness of breath *n* (%)	1 (2.7)	1 (5.3)	0 (0.0)	>0.99

#### Hospitalization and surgery costs

There were no statistical differences in the overall mean costs of OR and anesthesia between Group N and Group S ($23,009.20 ± 6,055.40 and $22,653.40 ± 7,920.70, respectively; *p* = 0.88). Likewise, the mean OR cost per minute was comparable among groups (Group N = $219.9 and Group S = $223.8; *p* = 0.38). In addition, the mean PACU costs for Group N were $4,764.10 ± 2,038.2 and $4,285.70 ± 1,697.6 for Group S (*p* = 0.44). Moreover, the mean total hospitalization cost was similar between groups ($33,225 and $34,520.90 for Group N and S, respectively; *p* = 0.66) (Table 4).

**TABLE 4:** Hospitalization and surgery costs.

Variables	Neostigmine (*n* = 19)	Sugammadex (*n* = 18)	*P*-value
OR costs, USD, mean (SD)	21,531.3 (5,690.6)	21,180.1 (7,473.8)	0.87
Anesthesia costs, USD, mean (SD)	1,477.9 (375.2)	1,473.3 (454.7)	0.97
OR and anesthesia, USD, mean (SD)	23,009.2 (6,055.4)	22,653.4 (7,920.7)	0.88
OR cost per minute of surgery, USD, mean (SD)	219.9 (13.18)	223.8 (13.1)	0.38
PACU costs, USD, mean (SD)	4,764.1 (2,038.2)	4,285.7 (1,697.6)	0.44
Total hospitalization costs, USD, mean (SD)	33,225 (7,457.2)	34,520.9 (10,327.7)	0.66

USD, United States dollar; OR, operating room; PACU, post-anesthesia care unit.

## Discussion

Our results indicate that patients undergoing outpatient surgeries and receiving sugammadex for moderate neuromuscular blockade reversal reached a TOF ratio ≥ 0.9 faster than patients receiving neostigmine/glycopyrrolate. This finding has been consistently reported in previous studies (17, 18, 24–29). However, we found no evidence of a significant reduction in OR readiness-for-discharge time as a result of a shorter time to reach a TOF ratio ≥ 0.9 in our patient population.

In an open parallel study, Sacan et al. (27) reported a significant reduction on the times to achieve TOF ratios of 0.7, 0.8, and 0.9 in patients receiving sugammadex when compared to edrophonium and neostigmine groups (*p* < 0.05). In addition, sugammadex administration has been associated with a faster deep neuromuscular blockade (1–2 PTC responses) reversal and a greater predictability to a TOF ratio of 0.9 within 5 min in comparison with neostigmine (98% versus 11%, respectively) (28, 29).

Grintescu et al. compared the recovery time in 34 patients undergoing laparoscopic cholecystectomy who received either sugammadex (2 mg.kg^–1^) or neostigmine (50 μg.kg^–1^) for moderate neuromuscular blockade reversal. Faster recovery time, defined as the time between the administration of the reversal agent and the time of extubation, was associated with the use of sugammadex when compared with neostigmine (1.2 ± 0.8 versus 16.7 ± 6.9 min, respectively; *p* < 0.001). Moreover, authors reported that the total time spent in the OR was significantly lower in the sugammadex group when compared to neostigmine (64.4 ± 24.3 versus 80.3 ± 20.5 min, respectively; *p* < 0.05) (22).

In a recent meta-analysis, Carron et al. analyzed data from 6 studies including a total of 518 patients undergoing different laparoscopic procedures in order to determine the efficacy of sugammadex on reducing the OR readiness-for-discharge time. Authors reported a significant association between sugammadex use and faster discharge from the OR to the PACU when compared with neostigmine (mean difference = 22.14 min, 95% CI (14.62, 29.67), *p* < 0.00001, *I*^2^ = 0%). Likewise, the time of PACU readiness-for-discharge to the surgical ward was significantly reduced in patients receiving sugammadex in comparison with those receiving neostigmine (*p* = 0.0469) (30). These results are consistent with previous meta-analyses, prospective, and retrospective studies (16, 25, 31, 32).

In our study subjects, neuromuscular blockade reversal was administered once surgeons started the laparoscopic ports closure. Additionally, reappearance of the fourth twitch in the TOF was required to administer neuromuscular blockade reversal in Group N. Compared to neostigmine/glycopyrrolate administration, Sugammadex was associated with reduced times from incision closure to extubation (4.5 versus 7 min), reversal administration to extubation (10.5 versus 13 min), and reversal administration to OR readiness-for-discharge (14 versus 15 min). However, none of these variables were statistically significant.

A faster reversal of the neuromuscular blockade allows for an early recovery of muscle tone and therefore, reduced incidence of postoperative complications. Stimulation of muscle spindles during reestablishment of muscle tone results in activation of spinal motoneurons and increases neural activity in the cerebral arousal centers (afferentation theory), especially in the reticular activity system (RAS) (33, 34). In a multicenter randomized clinical trial by Khuenl-Brady et al. (35), the incidence of general muscle weakness was comparable among patients who received sugammadex and those receiving neostigmine.

The overall incidence of postoperative complications in our study was 21.6%. Urinary retention and shortness of breath were reported in Group N only (5.4% and 2.7% of patients, respectively), whereas a higher incidence of postoperative nausea and vomiting was observed in Group S (22.22% versus 5.26%). Cholinesterase inhibitors (i.e., anticholinesterases) such as neostigmine remain the most frequently used drugs for neuromuscular blockade reversal. However, their effectiveness is limited in procedures where deep neuromuscular blockade is required. Moreover, these drugs must be combined with anticholinergic drugs (e.g., atropine, glycopyrrolate) in order to avoid undesired muscarinic effects including bradycardia, hypotension, bronchospasm, increased gastrointestinal motility, postoperative nausea and vomiting, and miosis (21, 36–39). In addition, anticholinergic drugs have an inhibitory effect on bladder contraction by antagonizing the postjunctional muscarinic receptors located in the detrusor muscle. This may increase the risk of postoperative urinary retention (40, 41). In our study, the two patients experiencing urinary retention (*n* = 2) were in Group N and required prolongation of their hospitalization until resolution (up to 48 h).

In contrast, sugammadex does not interfere with the acetylcholinesterase receptor system. In addition, the use of sugammadex has been linked to a faster and predictable reversal of any degree of neuromuscular blockade, reduced incidence of residual neuromuscular block and more efficient utilization of healthcare resources (12, 42). However, hypersensitivity reactions, cough, oral discomfort, increased partial thromboplastin time (PTT), severe bradycardia, and asystole have been also described after its administration (13, 39, 43, 44). Postoperative nausea and vomiting was the only adverse effect reported in Group S in our study (*n* = 4). Nevertheless, prolongation of the hospitalization was not necessary in any of these patients.

Cost-effective use of resources is paramount for every healthcare system in the world. An efficient use of surgical areas such as the OR and PACU is an essential component of any hospital budget structure (32). Our study population included patients undergoing ambulatory procedures under general anesthesia with a moderate to deep neuromuscular blockade throughout the surgery. Therefore, high doses of sugammadex or neostigmine/glycopyrrolate for neuromuscular blockade reversal were not required in our patient setting. An important body of evidence suggests that sugammadex may indirectly contribute to a significant reduction in costs by decreasing the PACU LOS, anesthesia emergence time, and the incidence of postoperative respiratory complications in comparison with neostigmine (16, 17, 26, 30, 45–47). In addition to reduce overall costs, sugammadex use has been associated with an increased OR turnover when compared to neostigmine (16). However, Deyhim et al. (18) reported that this saved time in the OR was not correlated with an increased workload and/or the number of surgical cases.

Different models have been described to assess perioperative and hospitalization costs. Static models include the costs of reversal agents and anesthesia care, labor costs (e.g., doctors, nurses, and OR time) and costs of hospitalization (17, 18, 48). In our study, we used the UB-04 hospital forms to retrieve the OR services, anesthesia, and recovery room costs. This form has been widely used in case-control and retrospective studies assessing in-hospital costs (49, 50). Mean anesthesia costs were similar between groups in our patient setting ($1,473.3 in Group S versus $1,477.9 in Group N; *p* = 0.97). Moreover, the OR costs per minute of surgery were comparable among groups (*p* = 0.38), being PACU mean costs slightly reduced in Group S ($4,285.7 versus $4,764.1; *p* = 0.44). Therefore, we found no evidence of a significant increase in in-hospital costs in patients undergoing outpatient surgeries who received sugammadex when compared to those receiving neostigmine.

Morbid obesity, procedure performed, anesthesia type, past medical history of hypertension and scheduled surgery duration have been identified as the main factors associated with a prolonged PACU LOS in outpatient care centers (51). In addition, intraoperative surgical training activities caused significant delays in the last stage of the surgery in our academic institution, which could have resulted in subsequent prolongation of the OR-to-PACU readiness-for-discharge times and PACU LOS, regardless of the type of procedure and randomization group. Additionally, extubation was performed before reaching a TOF ratio ≥ 0.9 in more than a half of our patients in Group N. This type of clinical practice is consistent with what has been discussed in current guidelines and recommendations (11) and may have had an important impact in our data.

De Robertis et al. (52) conducted a retrospective chart review in patients undergoing bariatric surgery to assess the recovery time after sugammadex or neostigmine administration and the healthcare cost impact of a faster recovery. The study concluded that the use of sugammadex was associated with a faster time to achieve a TOF ratio of 0.9 (*p* < 0.05) and an Aldrete score of 10 (*p* < 0.05), as well as a less duration in the OR theater occupancy (93.3 versus 116.6 min; *p* < 0.05) (52). Our study showed a slightly reduction in the length of anesthesia and the time elapsed from neuromuscular reversal administration to extubation in patients receiving sugammadex when compared to those receiving neostigmine.

Our study had some other limitations that are worth mentioning. First, the small sample size may increase the likelihood of a Type II error skewing the precision and power of our results. However, statistical power and sample size were calculated based on previous reports describing the differences in time to reach a TOF ratio ≥ 0.9 in patients receiving neostigmine or sugammadex. Second, our study only included outpatient surgical procedures and the results regarding the PACU and total hospital LOS, and costs, that may not reflect the impact of sugammadex in more complex surgeries with high-risk patients. Third, it is possible that sugammadex may impact further saving resources that were not considered in our study; the retrieved costs were calculated from insurance claims and not from a detailed breakdown of perioperative healthcare cost utilized in each subject. Lastly, the variability in the surgery type and surgical skills among surgical personnel in our academic center could have affected the time between incision closure and extubation, and/or the time between the neuromuscular blockade reversal administration and extubation, with a subsequent impact on the time elapsed from neuromuscular blockade administration to OR discharge.

## Conclusion

Sugammadex offers a significantly faster, predictable, and safer recovery profile from neuromuscular blockade than neostigmine in patients undergoing outpatient surgical abdominal procedures. Moreover, we found no evidence of increased OR or PACU costs associated with sugammadex administration in our patient setting. Considering the high variability of hospitalization and surgery costs around the United States, our cost analysis may potentially serve as a reference for future perioperative research in academic institutions.

## Data availability statement

The raw data supporting the conclusions of this article will be made available by the authors, without undue reservation.

## Ethics statement

The studies involving human participants were reviewed and approved by Institutional Review Board approval (Office of Responsible Research Practices) at The Ohio State University. The patients/participants provided their written informed consent to participate in this study.

## Author contributions

JF, NS, WA, MA-R, AU, and SB: study conception and design. JF, AE, OD, WA, JH, and AU: data collection. JF, ME-V, NS, MA-R, WA, JH, AU, and SB: data analysis and interpretation. MA-R: perform the analysis. JF, ME-V, AE, OD, NS, and AU: manuscript drafting. JF, ME-V, MA-R, WA, JH, AU, and SB: manuscript critical revision. All authors read and approved the final manuscript.
